# Microstructure and Properties of Cu-Ni-W-Si Gradient Coating on Copper Alloy by Laser Cladding

**DOI:** 10.3390/ma19132781

**Published:** 2026-06-30

**Authors:** Kaiyu You, Qi Zhong, Hanchang Ye, Yuxiang Jiang, Chenjiayue Ji, Haoran Ouyang, Pengyuan Zhai, Fengcheng Li, Zhenyang Cai

**Affiliations:** 1 School of Materials Science and Engineering, Central South University, Changsha 410083, China; 2 New Technology Promotion Institute of China Ordnance Industries, Beijing 100089, China; 3 Key Laboratory of Non-Ferrous Metal Materials Science and Engineering, Ministry of Education, Central South University, Changsha 410083, China

**Keywords:** copper alloy, Cu-Ni-W-Si coating, laser cladding, orthogonal experiment

## Abstract

To enhance the surface hardness and wear resistance of copper alloy workpieces, a Cu-Ni-W-Si gradient coating was fabricated on a Cu-Cr-Zr alloy substrate using coaxial powder-feeding laser cladding technology. Employing surface macroscopic morphology, flaw detection results, and cross-sectional microstructure as evaluation methods, along with the coating’s surface microhardness as a performance indicator, orthogonal experiments were sequentially conducted on the laser cladding process parameters for the Cu-Ni-10(W,Si) bottom layer and the Cu-Ni-20(W,Si) top layer. The optimized process parameters were identified as follows: a laser power of 4500 W (5000 W for the top layer), a scanning speed of 30 mm/s (60 mm/s for the top layer), and a scanning step of 2 mm. Subsequently, the phase composition and microstructure of the Cu-Ni-W-Si gradient coating were analyzed, and the microhardness distribution as well as the room-temperature friction and wear performance were evaluated. The results show that the coating achieves a hardness of 417 HV, which is 5.8 times higher than that of the substrate, and exhibits a wear rate of 3.52 × 10^−4^ mm^3^/Nm, corresponding to 49.1% of the substrate’s wear rate. The excellent performance of the coating is attributed to the favorable gradient metallurgical bonding between the coating and the substrate, as well as the presence of finely dispersed WSi_2_ high-hardness wear-resistant phases within the coating.

## 1. Introduction

Copper alloys, owing to their excellent electrical conductivity, thermal conductivity, and mechanical strength, are widely used in aerospace, electronic devices, and high-end manufacturing, and also serve as a key material for continuous casting mold crystallizers [1,2,3,4]. The Cu-Cr-Zr alloy, which combines good thermal conductivity, corrosion resistance, and low cost, has become a preferred material for the water-cooled inner wall of continuous casting crystallizers [5,6]. However, copper alloys inherently exhibit low surface hardness (typically only 50–150 HV) and poor wear resistance, and are prone to softening at elevated temperatures. These limitations severely restrict their long-term service performance under the complex and harsh conditions of high-temperature molten steel, leading to increased equipment failure rates and sharply rising maintenance costs [7,8,9]. To overcome this bottleneck, surface strengthening technologies have become a research focus.

Currently, mainstream surface coating technologies include electroplating, cold spraying, thermal spraying, and laser cladding [10,11,12,13]. Among these, laser cladding technology has achieved significant progress in the field of metal surface engineering [14]. It not only effectively enhances the interfacial bonding between the coating and the substrate but also imparts properties such as high strength, high hardness, wear resistance, and high-temperature stability to the surface, thereby preventing issues like cracking and spalling of the coating during high-temperature service. This provides a reliable technical approach for the high-performance surface modification of copper alloy crystallizer inner walls [15,16,17,18,19].

When fabricating copper alloy coatings via laser cladding, copper and its alloys exhibit high laser reflectivity and high thermal conductivity, along with poor wettability [20,21]. These characteristics result in insufficient energy being concentrated on the copper substrate surface to form a high-quality cladding layer. Moreover, the high reflectivity of the laser beam can cause damage to the laser device itself [22,23,24]. Therefore, exploring the laser cladding process parameters for copper alloy surfaces is particularly critical. Furthermore, the significant difference in thermophysical properties between the copper alloy and the coating material can readily induce interfacial thermal stress concentration and cracking [22,23,24]. At present, the construction of functionally graded coatings with compositional gradients can strengthen the interfacial bonding between the coating and the substrate, mitigate the mismatch in their thermal expansion coefficients, and reduce thermal stress and residual stress [22,25,26]. However, the increase in the number of laser cladding layers leads to differences in the fabrication conditions for each layer. Taking a double-layer graded coating as an example, the bottom layer is clad directly onto the copper alloy surface, while the top layer is prepared on the bottom layer material; due to the difference in cladding substrates, their optimal process parameters also differ. At present, orthogonal experimental design is commonly employed to screen process parameters, but most studies rely solely on coating hardness for range analysis, often neglecting the independent optimization of process parameters for each individual layer within the graded coating [25,26]. Therefore, this study conducts orthogonal experimental optimization for both the bottom and top layers of the laser cladding coating.

Meanwhile, to reduce the thermophysical property mismatch between the copper alloy substrate and the coating, and to alleviate the interfacial thermal stress concentration and cracking tendency, Cu-Ni-W-Si is selected as the primary coating composition in this study. Ni, which has a crystal structure similar to Cu and is infinitely miscible with it [27], can enhance interfacial bonding strength and reduce coating cracking [28]. W and Si can compositionally designed according to the WSi_2_ phase, enabling the formation of stable WSi_2_ metal silicides during the laser cladding process, whose physicochemical properties lie between those of metals and ceramics. Compared with other pure ceramic reinforcing phases, these silicides not only exhibit better wettability with the copper matrix and a more compatible thermal expansion coefficient but also possess good high hardness, excellent high-temperature oxidation resistance and good compatibility with the Ni-based alloy, which can enhance the wear resistance of the coating while suppressing oxidative failure under high-temperature service conditions, thus offering significant application advantages in laser cladding modification [29,30,31].

In this study, the orthogonal experimental method was employed to investigate the effects of laser cladding power (P), scanning speed (V), and scanning step (H) on the quality of the Cu-Ni-W-Si gradient composite coating. Using the microhardness of the coating as the optimization index, the primary and secondary influences of each factor were clarified through range analysis, and the preparation processes for the Cu-Ni-10(W,Si) bottom layer and the Cu-Ni-20(W,Si) top layer were optimized. Subsequently, the phase composition of the coating was analyzed by X-ray diffraction, the internal microstructure and elemental distribution characteristics were observed using scanning electron microscopy and energy-dispersive spectroscopy, and finally, the microhardness and friction and wear properties of the coating were tested.

## 2. Experimental Methods

### 2.1. Substrate and Powder Material

The Cu-0.88Cr-0.18Zr (wt.%) alloy substrate used in this study was supplied by Jianghe Machinery Plant (Henan, China) and is characterized by good mechanical properties and high electrical conductivity. Its performance parameters are presented in Table 1.

**Figure 1:**
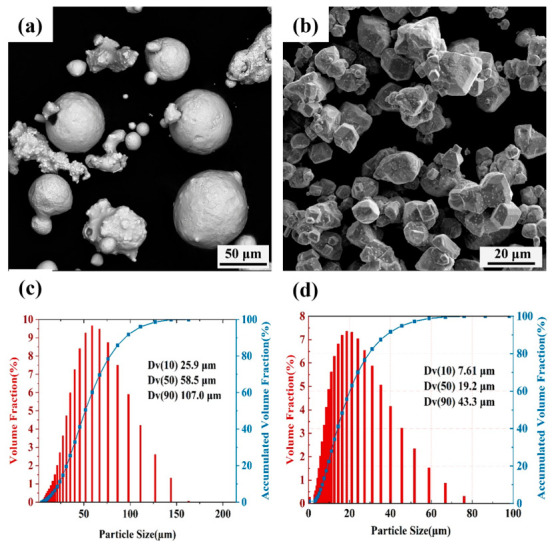
(**a**) Cu powder; (**b**) W powder; (**c**) particle size distribution of Cu powder; (**d**) particle size distribution of W powder.

The powders used for preparing the Cu-Ni-W-Si gradient coating, including elemental Cu, Ni, W, and Si powders, were supplied by Qinghe Top Materials Co., Ltd. (Qinghe, China). All powders used in this study were of analytical grade. The morphology and particle size distribution of the Cu and W powders are shown in Figure 1. The Cu powder particles are predominantly spherical, with some slight agglomeration observed on a portion of the particles. Their particle size distribution is relatively uniform and broad. The W powder particles are primarily crystalline in shape, featuring rough surfaces and sharp edges, and are significantly finer in size. Particle size distribution analysis of the powders prior to mixing was conducted using a MASTERSIZER 3000 laser particle size analyzer (Malvern Panalytical, UK). The results show that the W powder has a Dv (50) particle size of 19.2 μm, which is smaller than that of the Cu powder (58.5 μm).

### 2.2. Coating Preparation Process Flow

A center coaxial powder-feeding laser cladding method was employed to prepare the gradient coating on the surface of the Cu-Cr-Zr alloy. The laser cladding equipment used was a ZKZM-10000W laser cladding system (Xi’an, China), which utilizes a fiber laser. The powder feeding mode was center coaxial feeding, with argon as the shielding gas (Gas flow rate: 6 L/min). The laser cladding process parameters included laser power (4000–5000 W), scanning speed (30–120 mm/min), scanning step (1–3 mm), defocusing distance (0 mm), and powder feeding rate (18 g/min).

The preparation process flow for the Cu-Ni-W-Si gradient coating mainly includes raw material pretreatment, laser cladding of the bottom layer, and laser cladding of the top layer. The details are as follows:

(1) Raw material pretreatment

The Cu-Cr-Zr alloy was cut into specimens of dimensions 50 × 50 × 8 mm using wire electrical discharge machining. The specimens were sequentially ground with 200-mesh, 400-mesh, 600-mesh, and 800-mesh sandpaper, then ultrasonically cleaned in an ethanol solution for 15 min to remove surface oil and impurities, and finally vacuum-dried at 110 °C for 60 min. The prepared coating powders were mixed at a rotation speed of 50 r/min for 12 h, then vacuum-dried at 110 °C for 60 min, and subsequently placed into the powder feeder of the laser cladding system.

(2) Laser cladding of the bottom layer

The ZKZM-10000W coaxial powder-feeding laser cladding (Xi’an, China) equipment was selected. The orthogonal experimental method was employed to design and conduct experiments on the optimization factors (laser power (4000–5000 W), scanning speed (30–120 mm/min), and scanning spacing (1–3 mm)), using coating microhardness as the evaluation criterion to determine the optimal process parameters for laser cladding of the bottom layer on the copper alloy surface. The optimized parameters were used to prepare a dense and well-bonded Cu-Ni-10(W,Si) bottom layer. The loading table was preheated at 200 °C for 10 min before cladding, and the sample was placed in an insulated chamber for natural slow cooling after cladding.

(3) Laser cladding of the top layer

The main characterization techniques employed in this study include optical microscopy, X-ray diffraction, scanning electron microscopy, energy dispersive spectroscopy, and electron backscatter diffraction, which are primarily used to analyze the phase composition, microstructural morphology, and microstructure of the coatings.

Prior to microstructural observation, the coating samples were sectioned by wire electrical discharge machining and mounted, then sequentially ground with 400, 800, 1200, and 2000 grit SiC abrasive papers, followed by polishing with 2.5 μm diamond paste. The Cu-Ni-20(W,Si) top layer was cladded onto the bottom layer. Before cladding, the surface of the bottom layer was ground to remove surface oxides and oil contaminants. The orthogonal experimental method was used to optimize the preparation process parameters (laser power (4000–5000 W) and scanning speed (60–120 mm/min)) for the top layer. The optimized parameters were then employed to prepare the gradient coating (bottom layer + top layer). The loading table was preheated before cladding, and slow cooling was applied after cladding.

### 2.3. Coating Microstructure Characterization

A D/max 2500 VB (Tokyo, Japan) X-ray diffractometer was used to analyze the phase composition of the coating before and after performance testing. The diffractometer was equipped with a Cu target, operating at a voltage of 40 kV and a current of 250 mA. The scanning angle (2θ) ranged from 20° to 110°, with a scanning rate of 8°/min and a step size of 0.02°. A LEICA MC120 HD (Wetzlar, Germany) metallographic microscope was employed for preliminary observation of the cross-sectional microstructure of the coating, including coating thickness and bonding condition, providing reference for subsequent electron microscopic structural observation and compositional characterization. Prior to observation, the samples were ground, polished, and etched. The etchant used was a mixed solution of ferric nitrate, alcohol, and distilled water in a ratio of 12 g:10 mL:10 mL, and the etching time was 10 s (at 23 °C). A scanning electron microscope (SEM, TESCAN-MIRA4-LMH (Brno, Czech Republic)) equipped with an energy-dispersive spectrometer (EDS, GENESIS60S) was used to observe the microstructure morphology of the coating surface and cross-section, and EDS was employed for qualitative and semi-quantitative analysis of local compositions.

### 2.4. Coating Performance Testing

**Figure 2:**
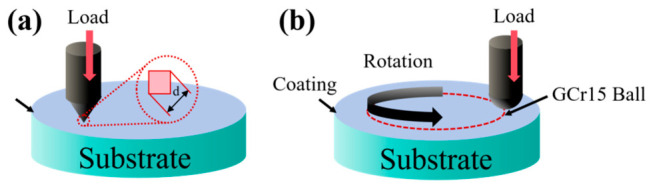
Schematic diagrams of coating performance testing principles: (**a**) principle of hardness testing; (**b**) principle of friction and wear testing.

The results of microhardness testing and friction-wear testing were used as the performance evaluation indicators for the Cu-Ni-W-Si gradient coating. Hardness, as one of the criteria for assessing coating quality, can effectively evaluate the deformation resistance of the coating when indented by a counter-material. According to the test method specified in the national standard ASTM C1327-15 [32], the cross-section of the coating was ground and polished to a mirror finish prior to testing. Subsequently, the cross-sectional hardness of the coating was measured using a Shimadzu HMV-2T (Kyoto, Japan) Vickers hardness tester under a load of 500 g with a dwell time of 15 s. Measurements were taken at intervals of 100 μm along the longitudinal direction of the coating cross-section, and the average value was calculated after three measurements. The principle of the hardness test is illustrated in Figure 2a.

A HT-1000 (Lanzhou, China) multifunctional friction and wear tester was used to conduct friction and wear tests on the substrate and the coating under room temperature conditions. The testing principle is illustrated in Figure 2b. According to the test conditions specified in the standard ISO 20808-2004, the experimental parameters were selected as follows: a load of 20 N, a rotational speed of 400 r/min, a counter-material of GCr15 steel ball with a diameter of 5 mm, and a circular wear track with a diameter of 6 mm for a duration of 30 min. The friction coefficient and wear loss of the materials were selected as the main indicators for evaluating wear resistance. To ensure the accuracy of the measured friction properties, the surfaces of the test samples were ground and polished flat using 2000-mesh sandpaper. Three samples were tested for each condition. The wear rate was calculated using the following Equation (1) [33]:

(1) $$Specific\;wear\;rate\;(m^{3}/Nm)=\frac{Wear\;Volume\;(m^{3})}{Normal\;load\;(N)\times Sliding\;distance\;(m)}$$

## 3. Results and Discussion

### 3.1. Laser Cladding Process for the Cu-Ni-10(W,Si) Bottom Layer

The optimization of the preparation process parameters for the bottom layer mainly focused on three factors: laser power (P), scanning speed (V), and scanning step (H). The prepared samples were labeled 1^#^ to 9^#^, and the areal energy density (E) corresponding to each set of process parameters is presented in Table 2. The factor and variable levels for the bottom layer laser cladding were as follows: laser power P (4000 W, 4500 W, and 5000 W), scanning speed V (30 mm/s, 60 mm/s, and 120 mm/s), and scanning step H (1 mm, 2 mm, and 3 mm). In this experiment, the laser spot diameter was set to 5 mm, and the Cu-Ni-10(W,Si) coating powder was used for cladding. Using the surface dye penetrant inspection results, cross-sectional microstructure quality, and microhardness as optimization criteria, the range analysis method was employed to evaluate the primary and secondary relationships between the laser cladding process parameters and the forming quality, and to obtain the optimal process parameters for the bottom layer preparation.

**Figure 3:**
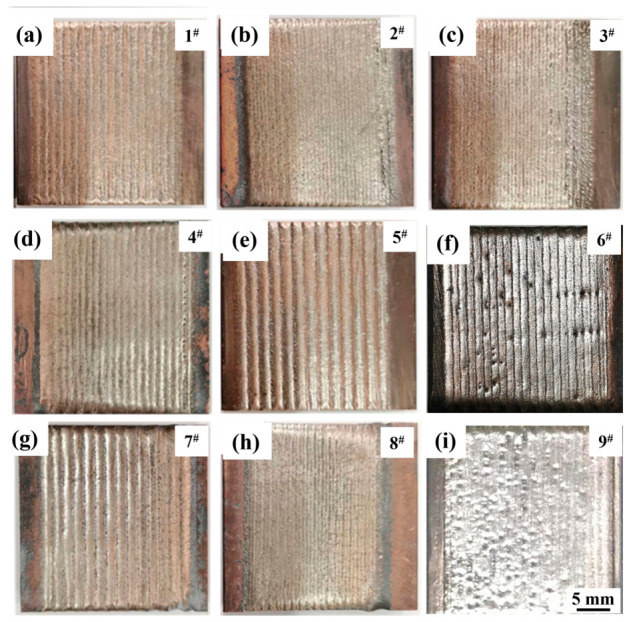
Macroscopic morphology of laser cladded coatings: (**a**–**i**) coatings prepared under process parameters 1^#^–9^#^, respectively.

According to the experimental parameters designed in Table 2, coatings with different process parameters (samples 1^#^ to 9^#^) were cladded on the Cu-Cr-Zr alloy surface. Their macroscopic morphologies are shown in Figure 3. All samples prepared under process parameters 1^#^ to 9^#^ exhibited good metallic luster on the surface. Except for samples 6^#^ and 8^#^, no obvious macroscopic cracks were observed on the coating surfaces. Sample 6^#^ exhibited numerous cladding discontinuities on the surface, which could easily become crack initiation sites for local cracking. For sample 8^#^, due to the high energy density of the laser beam, the temperature gradient during cooling was the largest, leading to a high tendency for crack formation. Overly wide overlapping between cladding tracks was clearly observed on the surfaces of samples 1^#^, 5^#^, and 7^#^. Because the overlap ratio between adjacent cladding tracks was too low, the molten metal could not be discharged in time after cladding, potentially leading to internal cracks and poor metallurgical bonding between cladding tracks. Samples 2^#^ and 3^#^ exhibited local discontinuities of cladding tracks at the initial stage of cladding on their surfaces, along with the presence of fine metal balls around the discontinuous tracks. For samples 2^#^ and 3^#^, at the initial cladding stage, the substrate surface temperature was low, resulting in poor wettability between the molten metal and the melt pool, making uniform spreading difficult. When the molten metal could not spread uniformly, it tends to solidify and contract on the surface into spheroidized balls of varying sizes, ultimately forming local discontinuities. Additionally, when the laser beam impinges on the melt pool, the kinetic energy of the laser beam is converted into surface energy of the fine balls, generating fine metal balls around the discontinuous tracks. As cladding proceeded further, heat accumulated on the surface, improving the wettability of the molten metal in the melt pool, ultimately resulting in relatively flat overlaps in the later stage of cladding. In Figure 3i, sample 9^#^ exhibited obvious pores on the surface. Because the areal energy density of the laser beam under the 9^#^ process was the lowest, the interaction between the laser beam and the powder/substrate was weak, resulting in poor wettability of the coating, deteriorated melt spreadability and fluidity, and ultimately the formation of pores. Comparing the above eight specimens, sample 4^#^ in Figure 3d exhibited good overlap between cladding tracks with high flatness on the surface, and no obvious macroscopic cracks or cladding track discontinuities were observed.

**Figure 4:**
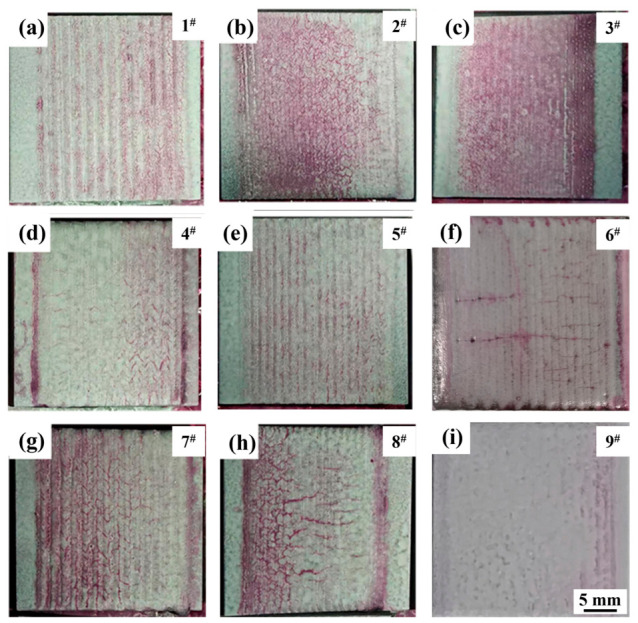
Dye penetrant inspection morphologies of laser cladded coatings: (**a**–**i**) coatings prepared under process parameters 1^#^–9^#^, respectively.

The coating surface may contain not only macroscopic cracks but also microscopic cracks. Macroscopic cracks can be directly observed on the sample surface, whereas microscopic cracks can only be identified using dye penetrant inspection. Figure 4 presents the dye penetrant inspection results of coatings prepared under different process parameters (samples 1^#^ to 9^#^), in which the continuous red areas indicate fine cracks. Samples 2^#^, 3^#^, 4^#^, 6^#^, 7^#^, and 8^#^ exhibit pronounced interlacing cracks, with samples 6# and 8# showing obvious longitudinal cracks. Owing to the rapid heating and rapid cooling characteristics of the laser cladding process, a large temperature gradient exists during melting and solidification, and the solidification time is short. Consequently, the cladded coatings are predominantly characterized by cold cracks [34,35]. Furthermore, copper alloys possess high thermal conductivity, which further increases the temperature gradient between the coating and the substrate. The thermal stress generated by this temperature gradient during cooling and solidification cannot be released in time and ultimately remains as residual stress within the coating [36]. When the residual stress exceeds a critical value, cracks initiate. Defects within the cladding layer are subjected to significant stress due to rapid heating and cooling, and pores act as stress concentration sites, becoming the first locations to crack [37]. Samples 2^#^ and 3^#^ do not exhibit continuous cracks at the initial stage of cladding; however, cracks appear in the coating during the later stage of cladding. Compared with the above seven specimens, samples 4^#^, 5^#^, and 9^#^ exhibit lower crack content on the surface. For sample 5^#^, due to excessive overlap, cracks are only present on the surface of the cladding tracks. In contrast, sample 9^#^, which has a low areal energy density, exhibits poor wettability on the surface, resulting in numerous discontinuities and pores. Sample 4^#^, subjected to a high laser beam energy density combined with the high thermal conductivity of the substrate, experiences significant stress, which ultimately leads to forming fine cracks on the coating surface.

**Figure 5:**
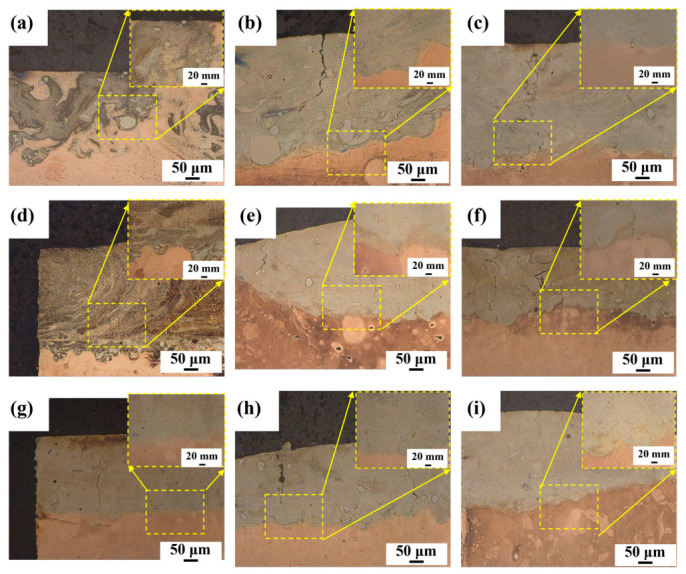
Cross-sectional and interfacial metallographs of laser cladded coatings: (**a**–**i**) coatings prepared under process parameters 1^#^–9^#^, respectively.

**Figure 6:**
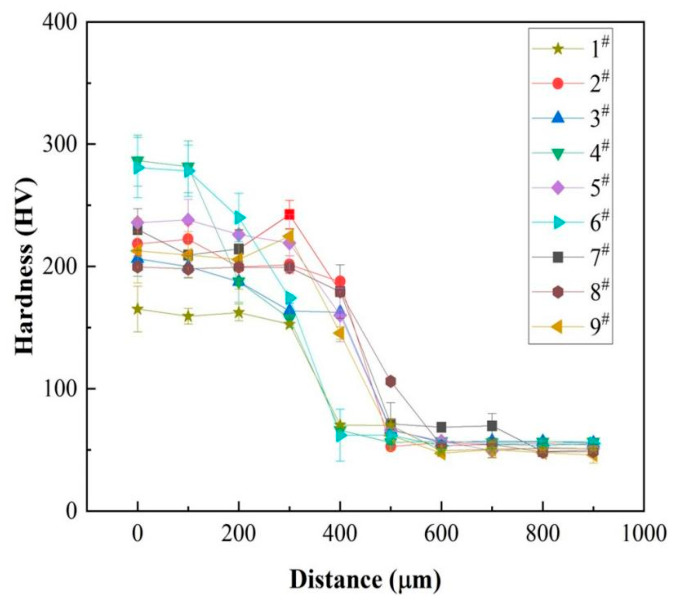
Cross-sectional hardness distribution of samples 1^#^–9^#^ from the orthogonal experiment and cross-sectional hardness indentation of sample 6^#^.

To further determine whether the surface cracks on the coatings extend into the interior, whether a bottom coating of good quality can be obtained after grinding, and whether a well-bonded interface is formed between the coating and the substrate, the cross-sectional microstructures of samples 1^#^ to 9^#^ were analyzed, and the resulting metallographic structures of the coating cross-sections are shown in Figure 5. Microhardness tests were conducted on samples 1^#^ to 9^#^, and the resulting microhardness curves of the coatings are presented in Figure 6. The average surface microhardness values of samples 1^#^ to 9^#^ are 165.1 HV, 218.4 HV, 206.4 HV, 286.5 HV, 235.8 HV, 280.8 HV, 230.1 HV, 199.7 HV, and 212.8 HV, respectively. Among these, samples 4^#^ and 6^#^ exhibit higher surface hardness (286.5 HV and 280.8 HV, respectively), while sample 1^#^ exhibits the lowest hardness (162.2 HV), with the hardness of the remaining samples falling between these values. From the microstructures shown in Figure 5, it can be seen that for samples 4^#^ and 6^#^, the higher energy density leads to an increased melting rate of W particles in the coating, forming more fine and dispersed phases. These dispersed phases hinder grain growth, thereby promoting grain refinement and enhancing the effect of fine-grain strengthening. In contrast, for sample 1^#^, due to the combination of low laser beam energy and a low overlap ratio, the excessively rapid cooling rate causes Marangoni forces within the melt pool to affect fluid flow [38], and the resulting segregation reduces the hardness of the coating.

The microhardness results from the orthogonal experiments were processed using range analysis, and the obtained analysis results are presented in Table 3. The bottom layer of the coating requires a certain degree of deformation resistance. Meanwhile, considering that fewer cracks on the surface and cross-section lead to higher stability, the primary and secondary order of factors and the optimal levels vary for each evaluation criterion. The main factors influencing microhardness are scanning step (H) and laser power (P), with the optimal level combination being P2V3H2. By comprehensively analyzing the surface macroscopic morphology, dye penetrant inspection results, and cross-sectional microstructure of the coatings, it was found that scanning speed (V) is the most sensitive factor affecting crack formation in the coating and has the least influence on microhardness. Consequently, P2V1H2 was determined as the optimal laser cladding process combination. Therefore, the optimized process parameters for the bottom layer are a laser power (P) of 4500 W, a scanning speed (V) of 30 mm/s, and a scanning step (H) of 2 mm.

### 3.2. Laser Cladding Process for the Cu-Ni-20(W,Si) Top Layer

During the preparation of the bottom layer, due to the high reflectivity of Cu to the laser beam, the actual energy incident on the substrate surface is much lower than the energy emitted by the laser. If the process parameters used for cladding the bottom layer were to be directly applied, the laser energy acting on the powder and the bottom layer would be too high, causing significant dilution of the bottom layer and increasing the tendency for segregation and cracking within the coating [39]. Therefore, when preparing the top layer on top of the bottom layer, the process parameters for the top layer need to be re-optimized to achieve the optimal energy density for laser–powder interaction and to produce a top layer with high cladding quality. After the optimal overlap step distance was determined, the laser power and scanning speed were subsequently optimized. The optimization parameters are listed in Table 4, and the prepared samples are labeled 1^#^ to 6^#^. The other cladding parameters were as follows: scanning step of 2 mm, laser spot diameter of 5 mm. The compositions of the bottom layer and the top layer were Cu-Ni-10(W,Si) and Cu-Ni-20(W,Si) coating powders, respectively.

**Figure 7:**
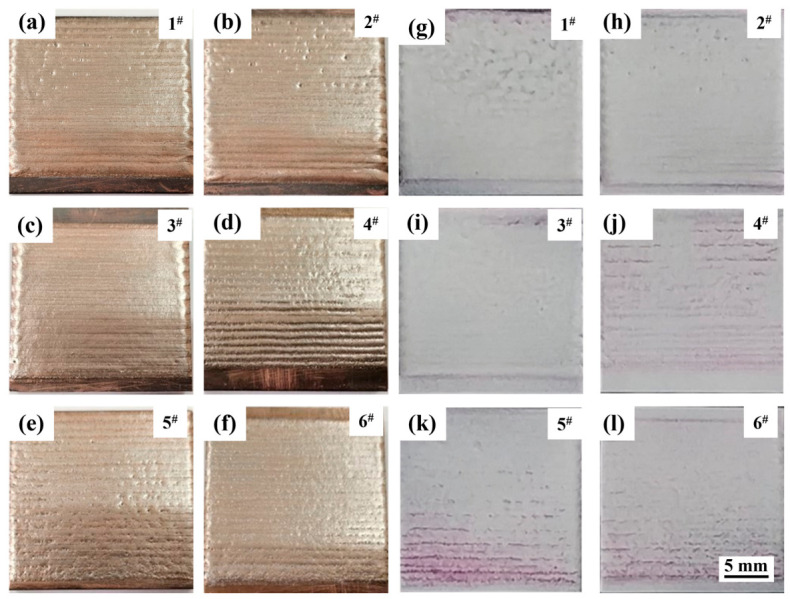
Cu-Ni-20(W,Si) coatings under different laser cladding processes: (**a**–**f**) macroscopic morphologies; (**g**–**l**) dye penetrant inspection results.

According to the experimental parameters designed in Table 4, Cu-Ni-20(W,Si) top layers with different process parameters (samples 1^#^ to 6^#^) were cladded onto the surface of the Cu-Ni-10(W,Si) bottom layer. The top layer samples are shown in Figure 7a–f. It can be observed that all samples 1^#^ to 6^#^ exhibit good metallic luster on the surface, with no obvious macroscopic cracks on the gradient coating surface. In Figure 7d, sample 4^#^ exhibits local overlapping discontinuity on the surface. Comparing Figure 7a,b with Figure 7d,e, samples 1^#^, 2^#^, 4^#^, and 5^#^ exhibit small pits on the surface, while samples 3^#^ and 6^#^ in Figure 7c,f show smooth surfaces without pits. The formation of such pits and discontinuities is mainly attributed to the poor wettability of the powder, which prevents it from spreading uniformly on the surface. As the energy density of the laser beam increases, the molten metal gains better wettability and spreads more easily [40]. Dye penetrant inspection was further employed to evaluate the quality of the cladded surface and to ensure the stability of the bottom layer. The dye penetrant inspection results are shown in Figure 7g–l. As presented in Figure 7g–i, the gradient coating surfaces prepared under processes 1^#^, 2^#^, and 3^#^ exhibit no obvious cracks. However, in Figure 7j–l, local cracks appear in the initial cladding region of the gradient coating. Compared with the optimal preparation parameters for the bottom layer, the cladding process for the second layer significantly reduces the cracking tendency of the coating.

**Figure 8:**
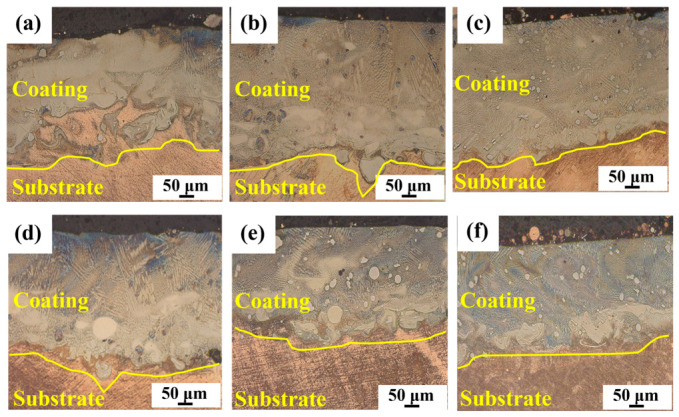
Cross-sectional metallographic structures of Cu-Ni-20(W,Si) coatings under different laser cladding processes for samples 1^#^–6^#^: (**a**–**f**) correspond to samples 1^#^–6^#^, respectively. (The interface between the coating and the substrate is indicated by yellow lines in the figure).

The denser the cross-sectional microstructure of the top layer coating, the better the forming quality of the top layer and the lower the cracking tendency. To obtain the metallographic structure of the gradient coating cross-section, the resulting microstructure is shown in Figure 8. The coating cross-sectional microstructures shown in Figure 8 are dense, with no cracks or pores observed. Before cladding the top layer, the oxide layer on the bottom layer surface must be removed by grinding. Consequently, when irradiated by a high-energy laser beam, downward dilution of the melt pool ultimately leads to a reduction in the thickness of the bottom layer. White particles are present in the cross-sectional microstructures of all top layers, being most pronounced in Figure 8d–f, while in Figure 8b,c, the white particles are fine and diffusely distributed, exhibiting a higher degree of grain refinement.

**Figure 9:**
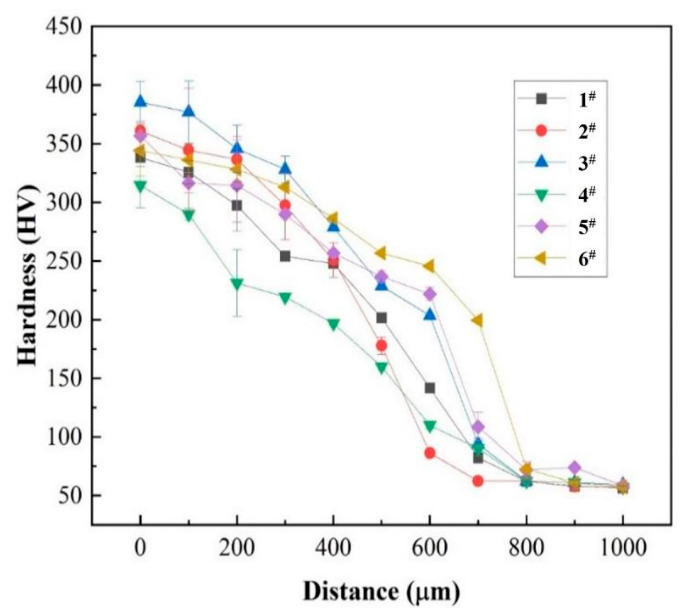
Hardness distribution of Cu-Ni-20(W,Si) coatings under different laser cladding processes and cross-sectional hardness indentation of sample 3^#^.

Figure 9 presents the cross-sectional hardness distribution of the top layer under processes 1^#^ to 6^#^ and the cross-sectional hardness indentation of sample 3^#^. The coating hardness exhibits a gradient distribution. The surface hardness values of the top layer under processes 1^#^ to 6^#^ are 345.6 HV, 361.0 HV, 385.6 HV, 320.2 HV, 356.7 HV, and 347.2 HV, respectively. Owing to the low areal energy density of the laser beam and the uneven distribution of internal precipitates, sample 4^#^ exhibits the weakest resistance to plastic deformation and the lowest hardness. Sample 3^#^ exhibits the highest surface hardness. Although the dilution effect from the substrate is relatively strong, the significant fine-grain strengthening effect counteracts the softening influence caused by substrate dilution. The more uniformly the precipitates are distributed, the more pronounced their effect on grain nucleation and growth, resulting in finer grain sizes and a more significant fine-grain strengthening effect.

The evaluation results from the orthogonal experiments on the gradient layer were processed using range analysis, and the obtained analysis results are presented in Table 5. Among the microhardness indicators, laser power (P) was identified as the primary influencing factor, and the optimal level combination was determined to be P3V1. This corresponds to the following process parameters: a laser power (P) of 5000 W, a scanning speed (V) of 60 mm/s, and a scanning step (H) of 2 mm.

### 3.3. Microstructure and Properties of the Cu-Ni-W-Si Gradient Coating

#### 3.3.1. Phase Composition of the Coating

**Figure 10:**
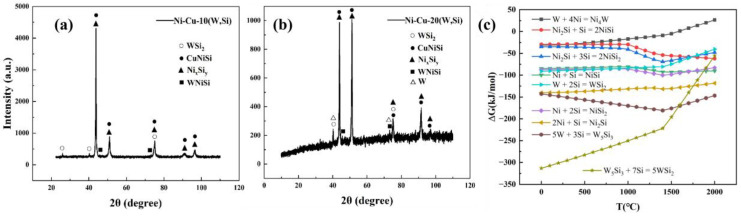
XRD diffraction patterns and Gibbs free energy curves: (**a**) XRD results of the Cu-Ni-10(W,Si) bottom layer; (**b**) XRD results of the Cu-Ni-20(W,Si) top layer; (**c**) Gibbs free energy curves for Reactions 2 to 10.

Figure 10a,b presents the XRD analysis results of the Cu-Ni-10(W,Si) and Cu-Ni-20(W,Si) coatings prepared by laser cladding on the copper alloy. Both coatings are composed of a CuNiSi solid solution, which may be attributed to the facts that Ni and Cu share the same FCC crystal structure and have similar lattice parameters, allowing the formation of an unlimited solid solution. In the Cu-Ni-10(W,Si) and Cu-Ni-20(W,Si) coatings, phases such as WNiSi, WSi_2_, and Ni_x_Si_y_ (including the stable phase NiSi_2_ and the metastable phases NiSi and Ni2Si of the Ni-Si compound) were also observed. Additionally, diffraction peaks corresponding to the W phase were detected in the Cu-Ni-20(W,Si) coating.

Through thermodynamic calculations, the Gibbs free energy values of various phases in the system can be obtained, enabling the prediction of possible chemical reactions, the determination of reaction directions, and the assessment of the stability of the resulting products. The possible reactions among the elements Ni, Cu, W, and Si in this experiment are primarily as follows:

2Ni + Si = Ni_2_Si (2)

Ni + 2Si = NiSi_2_ (3)

Ni_2_Si + Si = 2NiSi (4)

Ni_2_Si + 3Si = 2NiSi_2_ (5)

Ni + Si = NiSi(6)

5W + 3Si = W_5_Si_3_ (7)

W_5_Si_3_ + 7Si = 5WSi_2_ (8)

W + 2Si = WSi_2_ (9)

W + 4Ni = Ni_4_W (10)

To determine the difficulty of silicide formation, the Gibbs free energy data of the relevant reactions of each phase in the system were calculated using the thermodynamic calculation software HSC 9.0, and the calculation results were plotted as shown in Figure 10c. The Gibbs free energies for the reactions forming silicides such as WSi_2_ and W_5_Si_3_ are all negative, indicating that the above reactions can proceed under the temperature range of 0–2000 K. In addition to the formation of W_x_Si_y_ compounds (W_5_Si_3_ and WSi_2_), the Ni element added to the coating also exhibits a high affinity for Si, with its Gibbs free energies also being negative, suggesting that reactions may produce NiSi_2_, Ni_2_Si, and Ni_3_Si. Furthermore, during the laser cladding process for the gradient coating, the solidification cooling time is short, and the cladding of the gradient layer imposes a tempering treatment on the microstructure of the subsequent layer. Therefore, the thermodynamic calculation results merely represent the reaction outcomes among elements under ideal conditions, which can serve as a reference for the actual reaction products.

#### 3.3.2. Microstructure of the Coating

**Figure 11:**
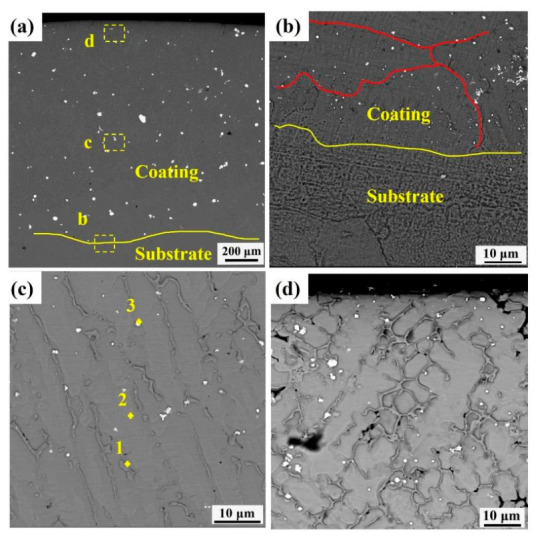
SEM images of different regions of the Cu-Ni-10(W,Si) coating: (**a**) cross-section; (**b**) microstructure of region b; (**c**) microstructure of region c; (**d**) microstructure of region d. (The interface between the coating and the substrate is indicated by yellow lines and the grains grown along the substrate at the interface are indicated by red lines in the figure).

Figure 11 shows the microstructure morphology of the Cu-Ni-10(W,Si) coating. The coating exhibits a dense structure, and a metallurgical bond is formed between the substrate and the coating. Within the coating, not only fine white dot-like precipitates but also larger white particles are dispersed. As can be seen from Figure 11b–d, the grain structure from the bottom to the top of the coating sequentially consists of coarse grains, elongated cellular crystals, and fine dendritic crystal axes. At the bottom of the coating, residual heat from the substrate results in a smaller temperature gradient compared with that at the top of the coating, leading to a larger G/R ratio (where G is the temperature gradient and R is the growth rate) [40], consequently, larger cellular crystals are formed at the bottom. In the middle region of the coating, the temperature gradient decreases, the G/R ratio decreases, the grain size becomes smaller, and the grain structure consists of fine equiaxed crystals. At the top region of the coating, the temperature gradient is even larger, and the grains are further refined into fine equiaxed crystals. From the XRD pattern in Figure 10a and the elemental contents in Table 6, the white dots can be identified as WSi_2_ (Point 3). The cellular crystals are mainly composed of (NiCu) and Ni_x_Si_y_ (Point 2), because NiSi compounds are randomly distributed within the NiCu lattice, forming the CuNiSi phase. Owing to the high energy and chemical instability of grain boundaries, metastable phases (such as Ni_31_Si_12_ and Ni_2_Si) segregate at the grain boundaries, leading to the enrichment of Si element (Point 1).

**Figure 12:**
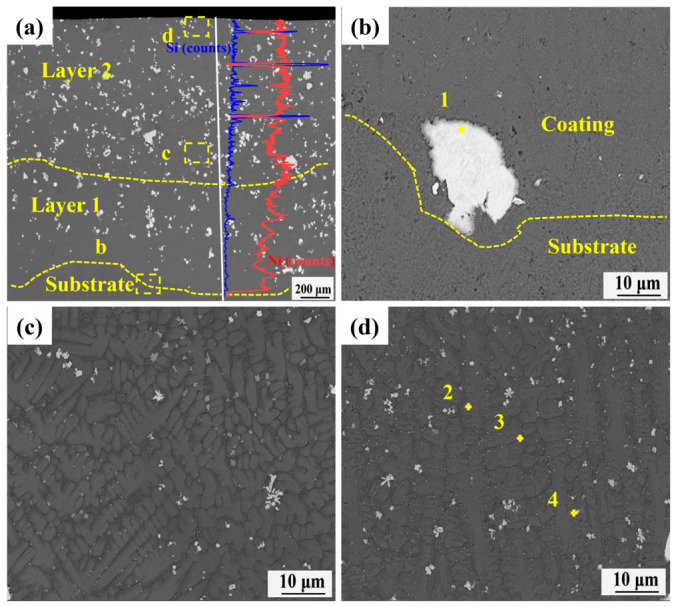
SEM images of different regions of the Cu-Ni-20(W,Si) layer: (**a**) cross-sectional microstructure and line scanning results (The interfaces between the substrate and the coating, as well as those between different coatings, are indicated by yellow dashed lines in the figure.); (**b**) microstructure of region b; (**c**) microstructure of region c; (**d**) microstructure of region d.

Figure 12 presents the microstructure morphology of the Cu-Ni-20(W,Si) coating. The coating exhibits a dense and defect-free structure, with dispersed precipitates and reinforcing phases of varying sizes. In region b at the bottom of the coating, the bonding interface between the coating and the substrate is not clearly defined, and white particles approximately 12 μm in size are observed. In region c of the coating, dendrites are clearly visible, along with finely dispersed dot-like phases approximately 1 μm in size, with precipitates mainly agglomerated near the grain boundaries. In region d at the top of the coating, dot-like precipitates ranging from approximately 500 nm to 100 nm are dispersed, and even smaller precipitates are distributed along the grain boundaries. Elemental analysis of the grains and precipitates in Figure 12 was performed, and the results are presented in Table 7. The large particles at the bottom (Point 1) are primarily composed of W, with a small amount of Si, and are inferred to be W particles that have undergone Si diffusion at their edges. Within the grains (Point 2), the composition is mainly NiCu with a minor amount of Si, inferred to be a CuNiSi solid solution. At the grain boundary shown as Point 3, Si enrichment is observed along with a small amount of W, inferred to be an enrichment site of unstable silicides. The fine dot-like precipitates have an atomic ratio of W to Si close to that of WSi_2_, and are therefore inferred to be WSi_2_. The variation in grain content within the microstructures of Cu-Ni-10(W,Si) and Cu-Ni-20(W,Si) is generally consistent. However, in Cu-Ni-20(W,Si), diffusion of Si into the W particles occurs in the large particles at the bottom, and the top region contains a greater number of fine precipitates, most of which are enriched along the grain boundaries.

Under low-temperature conditions, the atomic diffusion time is shortened, and the grain growth rate is restricted. Owing to the residual heat in the substrate, the temperature gradient at the top of the coating is relatively small, and the cooling rate is proportional to the temperature gradient, implying that the grain size decreases from the bottom to the top of the coating. The temperature gradient (G) and the growth rate (R) jointly determine the solidification structure of the coating, and the G/R ratio governs the solidification grain morphology [41]. The grain morphology transitions from large grains to cellular crystals to dendrites from the bottom to the top of the coating. On the one hand, silicides formed by the reaction of Ni and W with Si first appear as nuclei. Silicides represented by Ni_x_Si_y_ and WSi2 provide nucleation sites and promote heterogeneous nucleation. On the other hand, the addition of W and Si elements can restrict grain growth. The grain growth restriction factor (GRF), denoted as *Q*, is defined as $Q=m_{L}C_{O}(K-1)$, where *m_L_* is the liquidus slope, *C_O_* is the alloying element concentration, and *K* is the partition coefficient [42]. Low-melting-point components become enriched at the front of the liquid–solid interface because the higher-melting-point components begin to solidify. The increase in the initial solute concentration at the front of the solid–liquid interface promotes an increase in the GRF [41]. The grain size is inversely proportional to the GRF, and the addition of W and Si elements significantly enhances grain growth restriction [43,44]. Therefore, the crystal structure of the Cu-Ni-W-Si coating is regulated by the temperature gradient and the precipitates associated with the degree of grain refinement. The Cu-Ni-10(W,Si) coating is characterized by the presence of Ni_x_Si_y_ and WSi_2_ silicides at the grain boundaries, which effectively inhibit grain growth and form a typical laser cladding structure consisting of large grains, cellular crystals, and dendritic crystals. As the silicide content increases, nucleation sites increase, grain boundary obstruction is enhanced, and the degree of grain refinement increases.

#### 3.3.3. Microhardness and Friction-Wear Properties of the Coating

**Figure 13:**
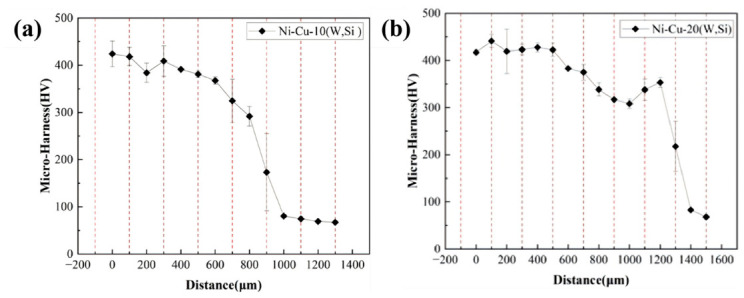
Cross-sectional hardness of the coatings: (**a**) Cu-Ni-10(W,Si) coating; (**b**) Cu-Ni-20(W,Si) coating.

Figure 13 presents the cross-sectional hardness distribution curves of the Cu-Ni-10(W,Si) and Cu-Ni-20(W,Si) coatings. As shown in Figure 13a for the Cu-Ni-10(W,Si) coating, the hardness increases abruptly at the interface, and the surface hardness reaches approximately 391 HV, which is 5.4 times higher than that of the substrate (72 HV). Figure 13b shows the cross-sectional hardness distribution curve of the Cu-Ni-20(W,Si) gradient coating. From the substrate to the coating surface, the hardness exhibits an increasing-decreasing-increasing trend, with the surface hardness reaching approximately 417 HV, which is 5.8 times higher than that of the substrate (72 HV).

**Figure 14:**
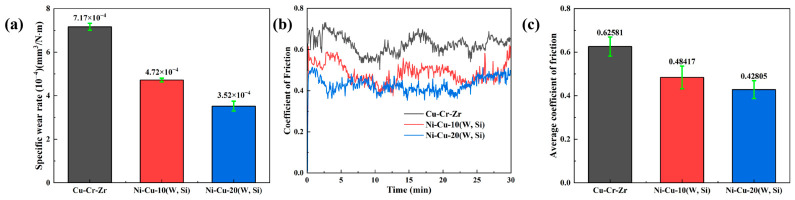
Friction and wear properties of the substrate, Cu-Ni-10(W,Si) coating, and Cu-Ni-20(W,Si) coating: (**a**) wear rates; (**b**) friction coefficient; (**c**) average friction coefficient.

Figure 14a presents the wear rates of the substrate, the Cu-Ni-10(W,Si) coating, and the Cu-Ni-20(W,Si) coating after 30 min of wear testing, with values of 7.17 × 10^−4^ mm^3^/Nm, 4.72 × 10^−4^ mm^3^/Nm and 3.52 × 10^−4^ mm^3^/Nm, respectively. It can be observed that the coatings all exhibit improved wear resistance compared with the substrate, with the Cu-Ni-20(W,Si) coating demonstrating particularly good wear performance. The weight losses of the Cu-Ni-10(W,Si) and Cu-Ni-20(W,Si) coatings are 65.8% and 49.1% of that of the substrate, respectively. The friction coefficient curves of the substrate, Cu-Ni-10(W,Si) and Cu-Ni-20(W,Si) coatings are shown in Figure 14b, with average friction coefficients of 0.62581, 0.48417 and 0.42805, respectively. The results show that the average friction coefficients of both coatings are significantly lower than that of the substrate, with the Ni-Cu-20(W,Si) coating exhibiting the lowest friction coefficient, demonstrating the best friction-reducing effect. Combined with the specific wear rate data, this coating achieves a simultaneous improvement in both wear resistance and friction-reducing performance.

#### 3.3.4. Coating Wear Morphology and EDS Analysis

**Figure 15:**
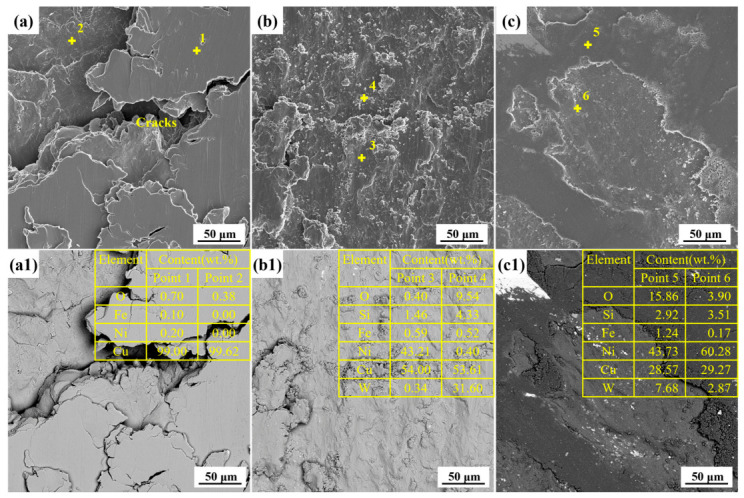
Wear morphologies and EDS elemental analysis results of the substrate and coatings: (**a**,**a1**) substrate; (**b**,**b1**) Ni-Cu-10(W,Si) coating; (**c**,**c1**) Ni-Cu-20(W,Si) coating. (**a**–**c**) are secondary electron (SE) images, and (**a1**–**c1**) are the corresponding backscattered electron (BSE) images.

Figure 15 shows the SEM surface morphologies of the Cu-Cr-Zr substrate, Ni-Cu-10(W,Si) coating, and Ni-Cu-20(W,Si) coating after friction and wear testing. Figure 15a clearly reveals the presence of large cracks on the worn surface of the Cu substrate, along with steps left by material detachment. As wear progresses, the cracks continue to propagate until the material on one side of a crack is detached from the substrate surface to form wear debris, leaving a step on the other side due to the resulting height difference. The EDS analysis results in Figure 15(a1) indicate that Point 2, which represents the freshly exposed rough surface after material detachment, exhibits a significantly lower oxygen content than the smooth surface at Point 1, which was flattened owing to its good plasticity. This is because the copper surface, with its inherently low hardness, is prone to continuous plastic deformation under contact stress, thereby leading to large-area tearing—a typical characteristic of adhesive wear. By contrast, the worn surfaces of the Ni-Cu-10(W,Si) coating and the Ni-Cu-20(W,Si) coating shown in Figure 15b,c are relatively smooth, indicating that the wear resistance of these coatings is markedly superior to that of the substrate and that a transition in the wear mechanism has occurred. In Figure 15b, although cracks and steps formed by material detachment are still present on the worn surface of the Ni-Cu-10(W,Si) coating, their dimensions are noticeably reduced. According to the EDS results in Figure 15(b1), the relatively smooth surface at Point 3 has a lower oxygen content than the rough surface at Point 4, and the oxygen content at Point 4 is significantly higher than that on the worn surface of the substrate. This suggests a shift in wear behavior from adhesive wear to oxidative wear. In Figure 15c, the worn surface of the Ni-Cu-20(W,Si) coating is even smoother, and the pits formed by the steps are shallower. The EDS analysis result (Figure 15(b1)) shows that the oxygen content at Point 5, representing the worn contact surface, reaches as high as 15.86 wt.%, indicating that oxidative wear is the dominant wear mechanism. The oxidation reaction on the surface, induced by frictional heating, leads to the formation of an oxide layer. Due to its relatively thin thickness and high brittleness, this oxide layer is easily detached, leaving behind shallow but relatively extensive pits.

## 4. Conclusions

The laser cladding process parameters on the surface of the Cu-Cr-Zr alloy were optimized through orthogonal experiments. A double-layer gradient coating consisting of a Cu-Ni-10(W,Si) bottom layer and a Cu-Ni-20(W,Si) top layer was successfully prepared. The surface morphology, microstructure, phase composition, microhardness, and friction and wear properties of the coating were systematically investigated and analyzed. The main conclusions are as follows:

(1) Through range analysis of the microhardness results from the orthogonal experiments, the optimal preparation process parameters were determined as follows: a laser power of 4500 W (5000 W for the top layer), a scanning speed of 30 mm/s (60 mm/s for the top layer), and a scanning step of 2 mm.

(2) After process optimization, the gradient coating exhibits good interfacial bonding and a dense, defect-free microstructure. Its structural architecture consists of NiCuSi solid solution cellular crystals, intergranularly distributed Ni_x_Si_y_ phases (NiSi_2_, NiSi, and Ni_2_Si), as well as finely dispersed WSi_2_ phases.

(3) The microhardness and room-temperature friction and wear performance of both the bottom and top layers are significantly enhanced. Their microhardness values reach 391 HV and 417 HV, respectively, which are 5.4 and 5.8 times higher than that of the substrate (72 HV). Their wear rates are 4.72 × 10^−4^ mm^3^/Nm and 3.52 × 10^−4^ mm^3^/Nm, corresponding to 65.8% and 49.1% of the substrate wear rate (7.17 × 10^−4^ mm^3^/Nm), respectively.

## Figures and Tables

**Table 1:** Performance parameters of Cu-Cr-Zr alloy.

Property	Hardness (HV)	Electrical Conductivity (IACS)	Yield Strength (MPa)	Tensile Strength (MPa)
Value	72	83	420	475

**Table 2:** Orthogonal array and areal energy density for laser cladding process parameters of the Cu-Ni-10(W,Si) bottom layer.

Sample No.	P (W)	V (mm/s)	H (mm)	E (W.mm^–2^)
1^#^	4000	30	3	8.9
2^#^	4000	60	2	6.7
3^#^	4000	120	1	6.7
4^#^	4500	30	2	15
5^#^	4500	60	3	5
6^#^	4500	120	1	7.5
7^#^	5000	30	3	11.1
8^#^	5000	60	1	16.7
9^#^	5000	120	2	4.1

**Table 3:** Range analysis of orthogonal experiment results for the Cu-Ni-10(W,Si) bottom layer.

Test No.	P (W)	V (mm/s)	H (mm)	Z (HV)
1^#^	4000	30	3	165.1
2^#^	4000	60	2	218.4
3^#^	4000	120	1	206.4
4^#^	4500	30	2	286.5
5^#^	4500	60	3	235.8
6^#^	4500	120	1	280.8
7^#^	5000	30	3	230.1
8^#^	5000	60	1	199.7
9^#^	5000	120	2	212.8
$\bar{Z_{j1}}$	196.6	227.2	229.0	
$\bar{Z_{j2}}$	267.7	218.0	239.2	
$\bar{Z_{j3}}$	214.2	233.3	210.3	
R_j_	71.1	15.4	28.9	
Primary and secondary order of factors	P > H > V			
Optimal combination	P2	V3	H2	

**Table 4:** Orthogonal array and areal energy density for laser cladding process parameters of the Cu-Ni-20(W,Si) top layer.

Sample No.	P (W)	V (mm/s)	E (W.mm^–2^)
1^#^	4000	60	6.7
2^#^	4500	60	7.5
3^#^	5000	60	8.3
4^#^	4000	120	3.3
5^#^	4500	120	3.75
6^#^	5000	120	4.2

**Table 5:** Range analysis of orthogonal experiment results for the top layer.

Test No.	P (W)	V (mm/s)	Z (HV)
1^#^	4000	60	345.6
2^#^	4500	60	361.0
3^#^	5000	60	385.6
4^#^	4000	120	320.2
5^#^	4500	120	356.7
6^#^	5000	120	347.2
$\bar{Z_{j1}}$	332.9	364.1	
$\bar{Z_{j2}}$	358.9	341.4	
$\bar{Z_{j3}}$	366.4		
R_j_	33.5	22.7	
Primary and secondary order of factors	P > V		
Optimal combination	P3	V1	

**Table 6:** EDS results for Figure 11 (at.%).

Point	Ni	Cu	W	Si
1	35.3	59.4	0.2	5.1
2	35.7	61.2	0.3	2.8
3	30.7	23.7	12.4	33.2

**Table 7:** EDS results for Figure 12 (at.%).

Point	Ni	Cu	W	Si
1	0.2	3.6	90.2	6.0
2	56.5	35.2	1.6	6.7
3	62	12.5	7.3	18.2
4	37.2	7.4	17.2	38.2

## Data Availability

The data presented in this study are available on reasonable request from the corresponding author.
